# Latest Development in Multiple Myeloma

**DOI:** 10.3390/cancers12092544

**Published:** 2020-09-07

**Authors:** Yoichi Imai

**Affiliations:** Department of Hematology/Oncology, IMSUT Hospital, The Institute of Medical Science, The University of Tokyo, Tokyo 113-8654, Japan; imaiyo-tky@umin.ac.jp

**Keywords:** multiple myeloma, *KRAS*, AXL, miRNAs, proteasome inhibitor, immunotherapy, CAR T-cell therapy, ASCT, MRD, long-lived plasma cells, hepatitis B virus, chronic pain

Specialists in the field of multiple myeloma (MM) research have written a series of 12 articles (2 original articles, 10 reviews) in the Special Issue “Latest Development in Multiple Myeloma”. MM is an incurable hematologic malignancy derived from B cells [1,2]. Introduction of autologous stem cell transplantation (ASCT) and novel chemotherapeutic drugs such as proteasome inhibitors (bortezomib) and immunomodulatory drugs (IMiDs) such as thalidomide, lenalidomide, and pomalidomide have improved the survival rates of MM patients. However, a large number of patients suffer from relapsed/refractory MM, and there is a need for novel therapies for these MM patients.

The articles in this Special Issue, “Latest Development in Multiple Myeloma”, provide insights into understanding the biology and therapeutic innovations in the field of MM.

Point mutation in *KRAS* is one of the most frequently detected mutations in MM; however, no data determining the impact of *KRAS* mutations on the survival of MM patients are available. Weißbach and colleagues analyzed the coding regions of *KRAS* using sequencing in 80 newly diagnosed MM patients, treated uniformly with induction therapy comprising bortezomib followed by high-dose chemotherapy and ASCT [3]. No *KRAS* mutations, including KRAS^p.G12A^ and exon-4 mutations KRAS^p.A146T^ and KRAS^p.A146V^, were correlated with patient survival. However, functional analysis of HEK293 cells overexpressing these *KRAS* mutants revealed the activation of MEK/ERK- and sustained PI3K/AKT-signaling in MM cells. These findings may be helpful in developing targeted therapies against *KRAS* mutations in MM patients.

Yan et al. reviewed the role of *AXL*, a member of the TYRO3/AXL/MERTK receptor tyrosine kinase family, in solid cancers and hematological malignancies [4]. *AXL* is involved in the regulation of cell survival, proliferation, angiogenesis, and immune modulation. In MM, *AXL* mediates cell dormancy and is a possible therapeutic target for releasing the MM cells from dormancy and sensitizing them to chemotherapy.

The non-coding regions of the genome have been paid considerable attention for their important role in cancer pathogenesis. Among the non-coding RNAs, microRNAs (miRNAs) are 19–25 bases in length and control gene expression through messenger RNA destruction or translation inhibition. In MM, several miRNAs such as miR-15a and miR-16 function as tumor-suppressing miRNAs, whereas miRNAs such as miR-21 and miR-221 function as oncogenes (oncomiRs). The role of miRNAs in MM and their potential in the prediction of MM prognosis and the development of novel MM therapies were reviewed by Handa et al. [5].

Bortezomib is the first-in-class proteasome inhibitor (PI), and carfilzomib is a next-generation PI, which selectively and irreversibly inhibits proteasome enzymatic activities. Ixazomib was the first oral PI to be developed, and in combination with lenalidomide and dexamethasone, it exhibits robust efficacy and a favorable safety profile in MM patients. These PIs, together with other agents, including alkylators, IMiDs, and monoclonal antibodies, have been incorporated into several regimens. The review by Ito summarizes the biological effects and results of clinical trials investigating PI-based combination regimens, including novel drugs [6].

In MM, immune surveillance is impaired; disruption of antibody production and function of T cells, natural killer cells, and dendritic cells is also observed. Immunotherapeutic interventions such as allogeneic stem cell transplantation and dendritic cell-based tumor vaccines can overcome this impairment; however, prolongation of patient survival is reported in limited populations of MM patients. Tamura et al. reviewed novel immunotherapies such as immunomodulatory drug-intensified monoclonal antibodies, chimeric antigen receptor (CAR) T cell therapy targeting B cell maturation antigen (BCMA), antibody drug-conjugates, and bispecific antigen-directed CD3 T cell engager targeting [7]. 

Hosen reviewed CAR T cell therapy among novel immunotherapies and described several molecules other than BCMA as targets of CAR T cell therapy [8]. In particular, his group discovered activated integrin β_7_ as a specific target against MM for CAR T cell therapy [9]. MM-specific monoclonal antibodies (mAbs) were searched in more than 10,000 clones of mAbs; MMG49 was found to specifically bind to MM cells, but not to CD45^+^ normal leukocytes, in the bone marrow (BM) of most MM patients. MMG49 reacts with integrin β_7_, adopting the activated conformation and constitutively activating integrin β_7_ in MM cells. They generated CAR T cells using the antigen recognition domain of MMG49, and the CAR T-cell therapy was effective in a MM xenograft mouse model. The group plans to perform clinical trials using this CAR T cell therapy.

ASCT in relatively young MM patients without severe cardiac and pulmonary comorbidities is a standard therapeutic strategy, and there is a strong need to establish prognostic factors for patients treated with ASCT. In a retrospective study by Ozaki et al., MM patients treated with ASCT without maintenance therapy were analyzed to assess the impact of normal immunoglobulin (Ig) recovery on clinical outcomes [10]. Among the 50 patients analyzed, 26 patients showed polyclonal Ig recovery, defined as normalization of all values of serum IgG, IgA, and IgM, 1 year after ASCT. The progression-free survival (PFS) and overall survival (OS) of patients with Ig recovery were longer than those of patients without Ig recovery. These results suggest that polyclonal Ig recovery after ASCT may be a useful prognostic marker to prevent overtreatment with maintenance therapy in MM patients who underwent transplantation.

Two reviews by Pinto et al. and D’Agostino et al. described novel approaches to cure MM, often considered as an incurable hematological malignancy [11,12]. Conventional therapies for MM comprise novel drugs such as PIs, IMiDs, histone deacetylase inhibitors, and mAbs. Although these therapies improve survival outcomes, most patients have a MM relapse or become refractory due to drug resistance. The review by Pinto et al. focuses on the causes of drug resistance and identification of novel therapeutic targets to overcome drug resistance in MM [11]. The review by D’Agostino et al. analyzes the results of clinical trials involving combination therapies using new-generation drugs for high-risk smoldering MM and newly diagnosed MM [12]. In addition, they describe minimal residual disease (MRD)-driven therapeutic strategies for MM patients.

MM cells are derived from plasma cells (PCs); of the PCs, long-lived plasma cells (LLPCs) have the capacity to survive for very long periods. MM cells and LLPCs likely share extrinsic and intrinsic survival programs. Utley et al. reviewed the survival mechanisms of LLPCs to obtain insights regarding therapeutic strategies at the time of diagnosis of MM relapse [13]. Soluble factors and cellular partners in the bone marrow microenvironment constitute extrinsic survival signals; increased autophagy, metabolic fitness, unfolded protein response (UPR), and enhanced responsiveness to endoplasmic reticulum (ER) stress constitute intrinsic programs required for LLPC survival. These LLPC survival mechanisms are targets of the main MM drugs, including proteasome inhibitors (bortezomib), steroids (dexamethasone), and IMiDs (lenalidomide).

This Special Issue includes two reviews describing the complications associated with MM [14,15]. The reactivation of hepatitis B virus (HBV) during or after cytotoxic chemotherapy is a well-known complication in patients with hematological malignancies. Delays in detection of HBV reactivation and initiation of antiviral therapy in patients with hematological malignancies can cause severe hepatitis and life-threatening fulminant hepatitis. With the introduction of novel therapies for MM, the number of reported cases of HBV reactivation among MM patients has gradually increased. There is a preventive strategy for HBV reactivation in patients with malignant lymphoma; however, it is yet to be established in patients with MM. On reviewing the Japanese nationwide retrospective survey of HBV reactivation in MM patients, Tsukune et al. found that HBV reactivation in MM patients is not rare and that ASCT should be considered as a risk factor [14]. Approximately 20% of MM patients show HBV reactivation 2 years after the initiation of therapy, unlike malignant lymphoma. The long-lasting reception of therapy in most MM patients may explain the late onset of HBV reactivation in MM. A common symptom in patients with MM is chronic pain, and the review by Coluzzi et al. focuses on this symptom [15]. Chronic pain in MM patients can be of multiple types, including pain associated with myeloma bone disease (MBD), therapy-induced peripheral neuropathy such as bortezomib-induced pain, and post-herpetic neuralgia due to reactivation of varicella zoster virus. The review describes the physiopathological mechanisms of bone pain, central sensitization, and pain chronification. It also emphasizes the importance of accurately assessing pain based on clinical examination and pain classification with the selection of the right analgesic option for the patient.

In conclusion, targeted therapies against *KRAS* mutations, *AXL*, and miRNAs will improve the treatment of MM. PI and immunotherapies play central roles in MM treatment, and CAR T-cell therapy will become a strong strategy in immunotherapies. Polyclonal Ig recovery after ASCT may suggest a good prognosis for transplant-eligible patients. MRD-driven and LLPC-targeted therapeutic strategies may be useful to make it possible to cure MM. Control of HBV reactivation and chronic pain will be an important issue in the management of MM patients. As a Guest Editor, I hope this Special Issue will be helpful to basic and clinical researchers in the field of MM. 
